# In Vitro and In Vivo Evaluation of the Fertilization Capacity of Frozen/Thawed Rooster Spermatozoa Supplemented with Different Concentrations of Trehalose

**DOI:** 10.3390/ani14243586

**Published:** 2024-12-12

**Authors:** Kristýna Petričáková, Martina Janošíková, Martin Ptáček, Filipp Georgijevič Savvulidi, Lukáš Zita

**Affiliations:** Department of Animal Sciences, Faculty of Agrobiology, Food and Natural Resources, Czech University of Life Sciences Prague, Kamýcká 129, Suchdol, 165 00 Praha, Czech Republic; janosikova@af.czu.cz (M.J.); ptacekm@af.czu.cz (M.P.); savvulidi@af.czu.cz (F.G.S.); zita@af.czu.cz (L.Z.)

**Keywords:** cryoprotectant, flow cytometry, gene reserve, sperm evaluation, trehalose

## Abstract

The objective of this study was to evaluate the impact of the supplementation of varying concentrations of the impermeable disaccharide trehalose on the in vitro and in vivo fertilization capacity of cryopreserved rooster spermatozoa in the original Czech Golden Spotted Hen breed. The control trehalose concentration was 0 mM, while TRE50 (50 mM), TRE100 (100 mM), and TRE200 (200 mM) were used as experimental trehalose concentrations. The kinematic and functional parameters of frozen/thawed spermatozoa were evaluated in vitro using mobile computer-assisted sperm analysis and a flow cytometer. The addition of 100 mM trehalose demonstrated the most favorable results for total (34.17%) and progressive (3.57%) motility after thawing. A statistically significant difference was found for these kinetic parameters compared to the other monitored concentrations. This experimental group was also found to have a significantly higher percentage of spermatozoa without plasma membrane or acrosome damage (33.37%) compared to the TRE50 group (30.74%; *p* < 0.05) and the TRE200 group (29.05%; *p* < 0.05). In vivo, artificial insemination was performed to verify fertilization ability. Hens (n = 40) were artificially inseminated twice (10 hens/treatment) with a 3-day interval between inseminations. In conclusion, the addition of 100 mM trehalose significantly improved total and progressive motility after thawing and preserved plasma membrane and acrosome integrity (*p* < 0.05). The fertilization rate of eggs fertilized with semen frozen with the addition of 100 mM trehalose was not significantly different from the other concentrations tested or the control group but was numerically higher (23.21% vs. 15.20% of fertilized eggs in this group).

## Simple Summary

The Czech Golden Spotted Hen is the only original Czech hen breed included in the National Genetic Reserve Program. The objective of this program is to preserve the genetic resources of native breeds in the Czech Republic through reproductive biotechnologies, such as artificial insemination or the cryopreservation of sperm. The objective of the present study was to investigate the impact of varying concentrations of the non-penetrating cryoprotectant trehalose on sperm fertilization capacity. This was achieved by assessing sperm functional and kinematic parameters, as well as fertility rates. The established methodology was used to optimize the cryopreservation procedure for the National Program of Genetic Reserves of the Czech Golden Spotted Hen breed.

## 1. Introduction

The freezing of semen represents a crucial procedure in breeding programs across diverse livestock species. Its objective is to facilitate the spread of genetic material throughout the population and to utilize genetically valuable animals. This procedure represents an important tool for the conservation of genetic resources in endangered species, with the objective of preserving a significant number of insemination doses (IDs) [1]. Semen cryopreservation is currently the only feasible method for ex situ management of avian genetic diversity. However, the fertilization rate of poultry inseminated with frozen semen is dramatically lower than that of fresh semen. The sex cells of male birds are very sensitive to various types of manipulation, especially at low temperatures. For that reason, increasing the fertility of sperm cells in all poultry species by improving the overall freezing process is of deep scientific interest [2,3].

It is well known that the composition of the extender, suitable cryoprotectants, and optimal freezing and thawing rates are important factors for successful semen cryopreservation [4,5]. To our knowledge, there are no standardized cryopreservation extenders for poultry semen. An important task of current research is to investigate extenders modified for cryopreservation of rooster spermatozoa. The utilization of commercial extenders, which are designed primarily for short-term cooling, has already been confirmed as a possible approach. In combination with previously tested commercial extenders, N-methylacetamide (NMA) at a concentration of 9% was identified as an effective cryoprotectant for ID modification for the Czech breed of hens [6].

In the selection of cryoprotective medium components, preference is currently given to the use of organic substances that have stabilizing and antioxidant properties in spermatozoa that are more physiological for them [7,8]. Sugars are known to stabilize membranes by interacting with the polar head groups of low-hydration plasma membrane phospholipids to form a glass (vitrification), lowering the membrane phase transition temperature of dry lipids and providing energy for semen during incubation [9]. Sugars can also maintain the osmotic pressure of diluents, induce cell dehydration, which reduces the negative effects of water flow across the sperm membrane during freezing [10], and reduce the incidence of ice crystal formation in semen [11].

Unlike other non-permeable polymers, disaccharide cryoprotectants are naturally occurring. Many authors have noted the exceptional properties of trehalose disaccharide as a non-toxic organic cryoprotectant due to its high hydration capacity compared to other sugars, which explains the excellent efficacy of trehalose as a bioprotectant [12,13]. Trehalose (α-D-glucopyranosyl-α-D-glucopyranoside), a lesser-known sugar, is produced by a wide variety of organisms, including bacteria, fungi, yeast, insects, plants, and some invertebrates that are capable of resisting the effects of dehydration or freezing [4]. Although it has many functions, it can withstand freezing in these organisms [14,15]. The structure of trehalose gives it some useful properties. It is a glucose dimer linked by an α-1,1-glycosidic linkage. Its acetal linkage prevents the reduction of C-1 in each monomer, which increases its stability at extreme temperatures and reduces its susceptibility to acid hydrolysis at low pH [16].

Trehalose is a non-permeable disaccharide that seems to protect cells by increasing the extender’s tonicity and stabilizing the plasma membrane, possibly due to direct interaction with the phospholipid polar head groups of membrane phospholipids [17]. Trehalose seems to be more efficient than other sugars for the protection of spermatozoa in cryopreservation media, and many authors have reported its beneficial effect on semen cryopreservation in different species, such as bull [18,19,20], buffalo [21,22], ram [9,23,24,25,26], goat [27,28,29], stallion [30,31], dog [10,32], boar [33], deer [34], rabbit semen [35,36], and hare [37]. The beneficial effect of trehalose supplementation on the in vivo fertility rate of rams, stallions, buffaloes, and hares has been confirmed in multiple studies [23,25,31,37,38,39].

There are only a limited number of reports available on the effect of trehalose on the quality of poultry sperm after thawing [40,41,42,43,44]. The addition of high concentrations of trehalose to the sperm extender provides the most effective protection in terms of post-thaw motility parameters, recovery rates, thermal resistance, and acrosome integrity. This disaccharide increases membrane fluidity before freezing, resulting in greater resistance of spermatozoa to freeze–thaw damage [3]. 

The aim of this study was to assess the effect of trehalose supplementation of semen extender on the functional parameters of rooster spermatozoa and their fertilization capacity after thawing using in vitro and in vivo assays.

## 2. Materials and Methods

### 2.1. Animals and Housing

All procedures performed with animals were in accordance with the Ethics Committee of the Central Commission for Animal Welfare at the Ministry of Agriculture of the Czech Republic (Prague, Czech Republic), and were carried out according to Directive 2010/63/EU for animal experiments. All investigations and handlings were conducted using normal breeder practices following official laws in the Czech Republic (Animal Protection Against Cruelty Act; Act No.246/1992 Sb). Five roosters of the Czech Golden Spotted Hen, all representatives of the important bloodline with a demonstrable Certificate of Fowl Origin, were included in this study. The average age of the roosters was 76 weeks. The males were kept in individual cages under controlled environmental conditions (air temperature = approx. 20 °C; air humidity = 50–60%) throughout the whole study in the Demonstration and Experimental Centre of the Czech University of Agriculture in Prague (50°07′47.6″ N 14°22′07.0″ E). The day/night photoperiod was 14/10 h during the experiment. The roosters were fed a compound mixture produced for the Czech University of Life Sciences in Prague (Sehnoutek a synové s.r.o.; 15.00% crude protein and 11.56 MJ/kg of metabolizable energy). Feed and water were available to the animals ad libitum throughout the experiment.

### 2.2. Semen Collection and Insemination Dose Processing

A two-week adaptation period was implemented to ensure the reliability and validity of the subsequent experiment. During this period, the ejaculate samples were subjected to continuous testing. The mean motility of fresh semen was found to be 89.34 ± 3.195%, with 80.91 ± 4.555% of spermatozoa exhibiting intact plasma membranes and acrosomes. It should be noted that definitive proof of the fertility of roosters is lacking. A more detailed and comprehensive account of the processes involved in ejaculate collection and processing can be found later in this chapter.

After the adaptation period, semen samples from the roosters were collected on a weekly basis throughout October and November 2022. The interval between subsequent collections was set at 2 to 3 days. The collection of semen was conducted using the dorso-abdominal massage technique, as described by Burrows and Quinn [45]. The collection was always performed by the same person at 8 am. The ejaculates were stored at 5 °C until processing. The volume and color of the ejaculate were evaluated macroscopically immediately after collection and recorded directly from the semen collection tube. The volume of semen was expressed in microliters (μL). The volumes of ejaculate obtained from each rooster exhibited slight variations, with the mean volume of ejaculate collected from each individual being 0.5 microlitres. All semen samples that met the initial requirements (the minimum value of spermatozoa concentration was set at 2 × 10^9^ cells/mL) were combined and diluted in a commercially available extender, Poultry Media^®^ (IMV Technologies, L’Aigle, France), which did not contain antibiotics. The mixed sample was prepared using ejaculate from at least four roosters on each occasion. Subsequently, the samples were divided into four groups, each containing a different concentration of trehalose: control (0 mM), TRE50 (50 mM), TRE100 (100 mM), and TRE200 (200 mM). All samples were supplemented with an NMA cryoprotectant at a defined concentration of 9% to a final concentration of 200 × 10^6^/mL cells [6,46,47].

**Figure 1:**
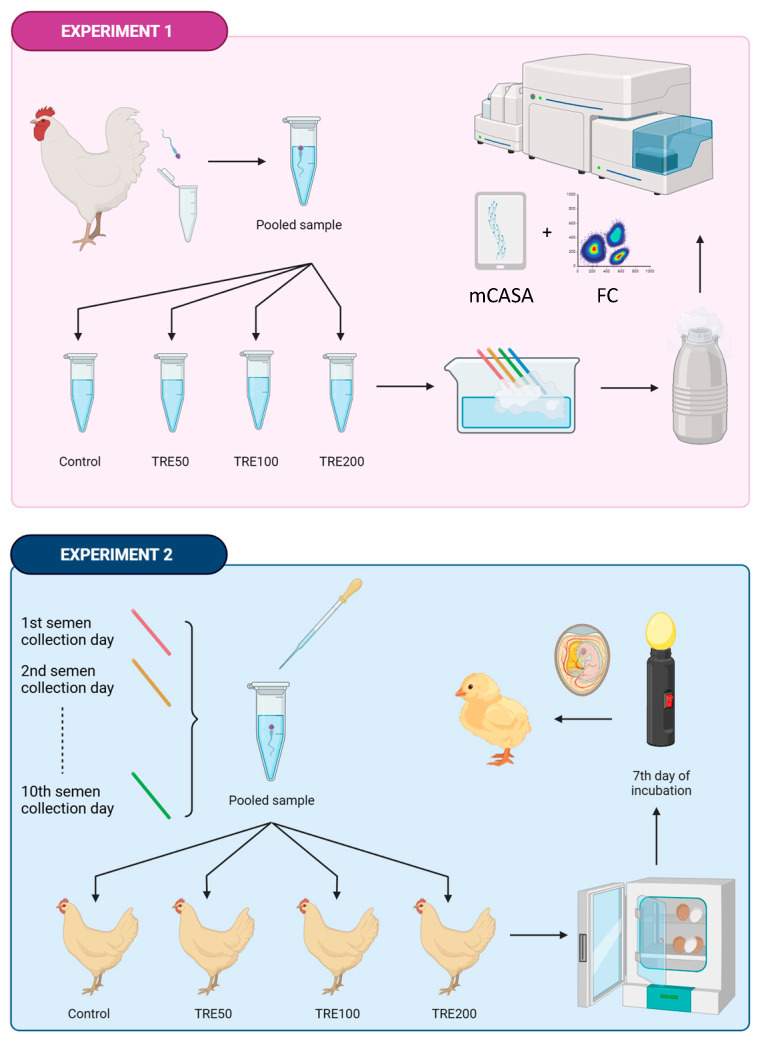
Study design. Experiment 1: collection of semen, processing of insemination doses, and evaluation of sperm functional parameters in vitro; Experiment 2: in vivo fertility assessment—insemination of hens, setting eggs in the hatchery, and assessment of fertility by candling the eggs after seven days of incubation. The figure was created using BioRender.com (accessed on 8 June 2024). Abbreviations: C—control group (no added trehalose); TRE50—samples containing 50 mM of trehalose; TRE100—samples containing 100 mM of trehalose; TRE200—samples containing 200 mM of trehalose; mCASA—mobile computer-assisted sperm analyzer; and FC—flow cytometry.

After equilibration at 5 °C for one minute [48], diluted semen samples were transferred into 0.25 mL French straws (IMV Technologies, L’Aigle, France) and sealed with sealing powder (IMV Technologies, L’Aigle, France). The IDs were frozen using liquid nitrogen vapor (the straws were placed 5 cm above the surface) for 10 min [49]. The freezing of the IDs was performed in a polystyrene mobile freezing box (commercial freezing box Minitube, GmbH, Tiefenbach, Germany). Subsequently, the straws were directly immersed in liquid nitrogen for the purpose of long-term preservation. Finally, the straws were transferred to a nitrogen tank (−196 °C) and kept at this temperature for three months before use. The design of this study is presented in Figure 1. 

### 2.3. Experiment 1: Evaluation of Sperm Functional Parameters

**Figure 2:**
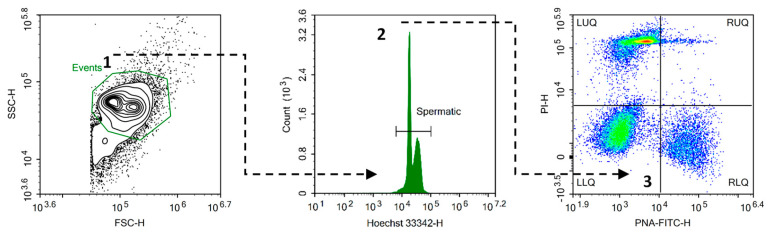
Flow cytometric gating strategy. A cluster of events was initially identified using a side scatter (SSC) versus forward scatter (FSC) bivariate histogram plot (1; green colored gate), followed by gating based on DNA content histogram (2). The gated spermatic event were divided into four groups according to the integrity of the plasma membrane and acrosome based on the density plot (3), identified by the intensity of the propidium iodide (PI) and Arachis hypogea lectin PNA (PNA-FITC) signals: LUQ—left upper quadrant (PI^+^/FITC-PNA^−^); RUQ—right upper quadrant (PI^+^/FITC-PNA^+^); RLQ—right lower quadrant (PI^−^/FITC-PNA^+^); and LLQ—left lower quadrant (PI^−^/FITC-PNA^−^). In this density plot, with density decrease, the color transitions from yellow over green to blue. The red color represents the highest density of events within the plot. Illustrative plots and histograms are shown. Dotted lines represent the gating hierarchy within the gating strategy.

The thawing procedure was performed in a water bath tempered at 5 °C for 100 s [46,50] to assess the quality of the insemination doses (IDs). Three IDs from each variation (modified extenders within the semen collection day) were thawed as described and pooled. Afterward, the exact aliquots of pooled sperm were assessed in three technical replicates, using computer-assisted sperm analysis and flow cytometry, as described below. All the data on technical replicates were averaged for subsequent statistical evaluation. The key functional spermatozoa parameters, such as the total motility (TMOT, %), progressive motility (PMOT, %), curvilinear velocity (VCL, μm/s), average path velocity (VAP, μm/s), linear velocity (VSL, μm/s), straightness (STR, %), and linearity (LIN, %) were assessed with a mobile mCASA analyzer (iSperm^®^, Aidmics Biotechnology, Taipei City, Taiwan) after thawing using the iSperm Poultry 5 (iSperm^®^, Aidmics Biotechnology, Taipei City, Taiwan) application. IDs were diluted with the extender that was used to dilute the semen before freezing to the mCASA manufacturer’s recommended concentration of 30 × 10^6^ sperm/mL. The diluted sample was dropped in a volume of 7 μL onto a special analysis disposable chamber that was fixed on the lens; then, the total motility was evaluated using the software. The plasma membrane and acrosome status were determined using flow cytometry assays. Thawed IDs for the flow cytometry assays were diluted with phosphate-buffered saline (Sigma Aldrich, St. Louis, MO, USA) to a final concentration of 10 × 10^6^/mL and then stained with a mix of fluorescent dyes at 38.5 °C for 15 min (in the dark). The following dyes were used in the mix (final concentrations given): 16.6 μg/mL Hoechst-33342 (H-342) to identify the presence of DNA; 13.3 μg/mL propidium iodide (PI) to detect plasma membrane damage; and 0.83 μg/mL PNA lectin from Arachis hypogea (PNA-FITC) to assess acrosome damage. H-342 and PI were purchased from Sigma Aldrich (St. Louis, MO, USA) and PNA-FITC from Thermo Fisher Scientific (Waltham, MA, USA). Flow cytometric parameters were assessed with a NovoCyte 3000^®^ flow cytometer (Acea Biosciences, Agilent, Santa Clara, CA, USA). For each sample, a minimum of 10 × 10^3^ events were analyzed. The flow cytometer was equipped with violet (405 nm, 50 mW) and blue (488 nm, 60 mW) lasers. The fluorescence was collected using the optical filters 445/45 (H-342), 530/40 (PNA-FITC), and 675/30 (PI). H-342 can be successfully excited with a violet (405 nm) laser [51]. NovoExpress software, v1.3.0 (Acea Biosciences, Agilent, Santa Clara, CA, USA) was used for data acquisition. The same software was used to analyze the acquired flow cytometry data. No compensation was required with the optical filter setup used. The gating strategy used for this research is presented in Figure 2. The events were initially identified using an SSC-H (side scatter) versus FSC-H (forward scatter) bivariate histogram plot. Spermatic events were identified based on the threshold and gating set with H-342 stain (DNA content). The sperm subpopulations were categorized as follows: PI^+^/FITC-PNA^−^ = spermatozoa population with damaged plasma membrane and slightly deteriorated acrosome located in the left upper quadrant; PI^+^/FITC-PNA^+^ = spermatozoa population with high damage to the plasma membrane and acrosome located in the right upper quadrant; PI^−^/FITC-PNA^+^ = spermatozoa population with intact plasma membrane and high acrosome damage located in the right lower quadrant; and PI^−^/FITC-PNA^−^ = spermatozoa population with both plasma membrane and acrosome intactness located in the left lower quadrant.

### 2.4. Statistical Evaluation

All statistical evaluations of the first experiment were performed in the SAS statistical program (SAS/STAT) v9.4. (SAS Institute Inc., Cary, NC, USA). The general linear model (GLM) procedure was used to analyze variables after a check of residual distribution (residual QQ plot) [6,52].

A statistical model with two fixed effects of the day of semen collection (10 classes) and cryoprotective media containing different concentrations of trehalose (4 classes) was applied to cover the variability among dependent variables. The following model equations were fitted for this estimation:

Y_ijk_ = μ + DAY_i_ + TRE_j_ + e_ijk_ (1)

where Y_ijk_—dependent variable (TMOT; PMOT; VAP; VCL; VSL; LIN; STR, PI^−^/FITC-PNA^−^; PI^+^/FITC-PNA^−^; PI^−^/FITC-PNA^+^; and PI^+^/FITC-PNA^+^); μ—mean value of the dependent variable; DAY_i_—fixed effect of the semen collection day (i = 27th October, n = 9; i = 31st October, n = 6; i = 3rd November, n = 6; i = 7th November, n = 9; i = 10th November, n = 12; i = 14th November, n = 12; i = 16th November, n = 12; i = 21st November, n = 12; i = 24th Novemeber, n = 12; and i = 28th November, n = 6); TRE_j_—fixed effect of the addition of cryoprotective media containing different concentrations of trehalose (j = without trehalose supplementation, n = 30; j = 50 mM trehalose supplementation, n = 18; j = 100 mM trehalose supplementation, n = 30; and j = 200 mM trehalose supplementation, n = 18); and e_ijk_—residual error.

The significance of the differences between the semen collection days and the individually modified extenders was tested using the Tukey–Kramer test. A significance level of *p* < 0.05 was used to evaluate the differences between groups.

### 2.5. Experiment 2: In Vivo Fertility Assessment

There was a total of 40 hens (mean age 63 weeks); 10 hens per treatment were inseminated with the frozen/thawed IDs obtained in Experiment 1. It is proposed that the lower prevalence of hens within the group was offset by the elevated number of fertilized eggs observed [53]. The relatively low number of monitored individuals is a consequence of the limited population size of this rare breed [1].

Insemination was performed twice a week (three days after the first insemination) during February 2023 [54,55]. A minimum of two cryopreserved straws from each day were thawed and mixed together to create a pooled insemination dose. Consequently, only the impact of trehalose concentration on fertilization rate was evaluated, as the influence of the collection day was eliminated.

Semen straws were thawed in ice water at 5 °C for 100 s [46,50]. The percentage of fertilized eggs laid when hens are inseminated while a hard-shelled egg is in uterus is lower than when inseminating hens that have just laid or hens without eggs in the oviduct [56]. Therefore, all inseminations were performed between 2 pm and 3 pm to avoid the presence of a shelled egg in the uterus according to the method of Burrows and Quinn [45]. A 250 µL artificial insemination glass pipette containing an appropriate amount of semen (250 µL containing 200 × 10^6^ sperm per female) was then inserted into the fallopian tube and semen was released at a depth of 2–4 cm [57]. 

The eggs were collected from the second day after the first insemination. The eggs were incubated at standard conditions in an automatic setter (Bioska Sedlčany s.r.o., Czech Republic). The eggs were candled on the 7th day of incubation for embryonic development and on the 18th day fertile eggs were transferred into the setter compartment. The chicks hatched on the 21st day of incubation were counted to calculate hatchability. The infertile eggs were broken open to confirm the absence of embryonic development.

### 2.6. Statistical Evaluation

The statistical analysis of the second experiment was performed using the general linear model (GLM) procedure to analyze a variable after a check of residual distribution (residual QQ plot) [6,52] in the SAS statistical program (SAS/STAT) v9.4. (SAS Institute Inc., Cary, NC, USA). Differences were considered significant when *p* < 0.05.

A statistical model with one fixed effect of cryoprotective media containing different concentrations of trehalose (4 classes) was applied to cover the variability among dependent variables. The following model equations were fitted for this estimation:

Y_ij_ = μ + TRE_i_ + e_ij_

where Y_ij_—dependent variable (fertilization rate); μ—mean value of the dependent variable; TRE_j—_fixed effect of the addition of cryoprotective media containing different concentrations of trehalose (j = without trehalose supplementation, n = 42; j = 50 mM trehalose supplementation, n = 62; j = 100 mM trehalose supplementation, n = 62; and j = 200 mM trehalose supplementation, n = 57); and e_ij_—residual error.

The significance of the differences between the semen collection days and the individually modified extenders was tested using the Tukey–Kramer test. A significance level of *p* < 0.05 was used to evaluate the differences between groups.

## 3. Results

### 3.1. Experiment 1: Motion Parameters and Membrane and Acrosome Integrity

**Figure 3:**
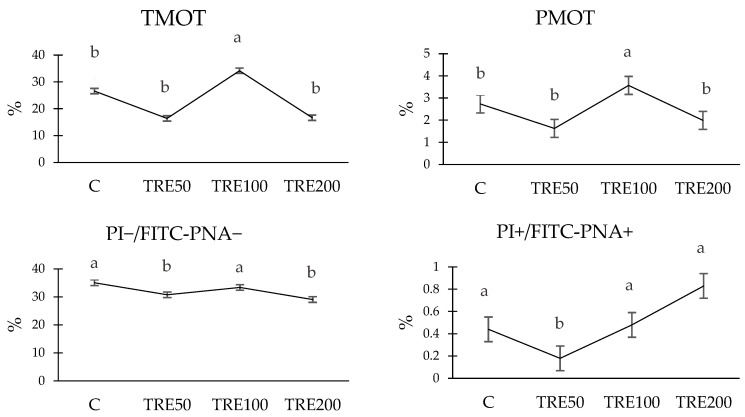
Kinematic and functional parameters of spermatozoa (LSM ± SEM) for which statistically significant differences were found between the groups tested (*p* < 0.05). Abbreviations: C—control group (no added trehalose); TRE50—samples supplemented with 50 mM of trehalose; TRE100—samples supplemented with 100 mM of trehalose; TRE200—samples supplemented with 200 mM of trehalose; TMOT—total motility; PMOT—progressive motility; PI^−^/FITC-PNA^−^—spermatozoa population with negative signal of plasma membrane or acrosome disruptions located in the left lower quadrant; and PI^+^/FITC-PNA^+^—spermatozoa population with high damage to the plasma membrane and acrosome located in the right upper quadrant. a,b Different letters indicate differences between groups within a row (*p* < 0.05).

The observed kinematic and functional parameters of spermatozoa were evaluated both before freezing (see Table S1, available in the Supplementary File) and after thawing. After thawing, statistically significant differences (*p* < 0.05) were observed in the variables TMOT and PMOT, as determined by assessing mCASA and FC in Experiment 1. The highest values were observed in the group that received the addition of 100 mM trehalose. Moreover, the highest proportion of spermatozoa with an intact plasma membrane and acrosome was observed in the group that received a 100 mM trehalose addition (see Figure 3). The results demonstrated that there was no statistically significant difference between the experimental group TRE100 and the control groups. Nevertheless, a significant difference (*p* < 0.05) was discerned in the other experimental groups, which received either a higher or a lower dosage of trehalose. No statistically significant difference was identified in the remaining kinematic and functional parameters. A list of all these variables can be found in Table S2, which is available in the Supplementary File. Additionally, all statistical models for the observed parameters of frozen/thawed sperm that were identified as significant are included in this table, with the exception of VSL.

### 3.2. Experiment 2: Fertility Evaluation

**Figure 4:**
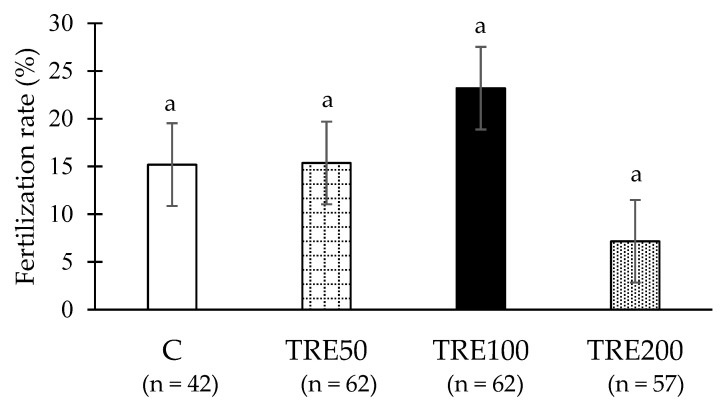
Fertilization rate after artificial insemination with the addition of different concentrations of trehalose (LSM ± SEM). Abbreviations: C—control group (no added trehalose); TRE50—samples supplemented with 50 mM of trehalose; TRE100—samples supplemented with 100 mM of trehalose; and TRE200—samples supplemented with 200 mM of trehalose. a The same letters indicate no statistically significant difference within a column (*p* < 0.05).

Of the 223 eggs collected and deposited, only 34 were successfully fertilized, representing a fertilization rate of 15.24% ± 6.067%. The highest fertilization rate was observed in the TRE100 group, with a success rate of 23.21%. Conversely, the least favorable outcome was observed when 200 mM trehalose was introduced, with a mere 7.16% of eggs successfully fertilized. There are notable discrepancies between the TRE100 and TRE200 groups, with a *p*-value of 0.06, just below the 0.05 threshold for statistical significance. The addition of trehalose did not result in a statistically significant impact on the fertilization rates (one-way ANOVA with *p* < 0.05; Figure 4).

## 4. Discussion

The oligosaccharide trehalose may benefit frozen spermatozoa by contributing to extracellular vitrification formation and reducing ice crystal production [9,58]. As a non-permeable disaccharide, trehalose can increase extracellular osmotic pressure, causing cell dehydration and decreasing the formation of lethal intra-cellular ice [59]. Moreover, trehalose can link with plasma membrane phospholipids, reorganize the plasma membrane, and make spermatozoa survive through cryopreservation [60]. Furthermore, trehalose can be integrated into the plasma membrane and prohibit the excessive dehydration of spermatozoa during cryopreservation, consequently reducing the physical damage caused by abnormal variations in cell volume [61,62]. In considering the above-mentioned beneficial effects of this disaccharide, we proceeded to study the effect of trehalose addition in the cryopreservation of rooster semen in the Czech hen breed.

### 4.1. The Effect of Trehalose Supplementation on the Kinematic and Functional Parameters of Spermatozoa In Vitro as an Indicator of Potential Fertility

The use of trehalose, as the only cryoprotectant, did not adequately preserve sperm quality after freezing/thawing, so the appropriate permeable cryoprotectant for the species and breed needs to be selected [47]. In various mammalian species, sperm cryosurvival was improved by combining permeable with non-permeable cryoprotectants. There have already been publications where trehalose has been used as an extender additive in different species [63]. To the best of our knowledge, in the protocols using trehalose concentrations of 100 mM and 70 mM, the best post-thawing semen parameters were observed in the ram [9,24,25], goat [28,62], boar [19,64], bull [65], and dog semen [10]. 

The non-permeable cryoprotectants trehalose and sucrose were studied in chicken semen cryopreservation [49,66]. Trehalose, unlike other disaccharides, has a positive protective action during the cryopreservation of chicken sperm [47,67]. In previous research on the freezability of goose sperm, the combination of 10% DMSO (Dimethyl sulfoxide) and 50 mM trehalose significantly improved motility, the acrosome integrity rate, and the plasma membrane integrity rate after thawing [68]. Adding 200 mM trehalose was successfully used for turkey semen cryopreservation [69]. However, it was harmful in combination with 6% DMF (N, N-Dimethylformamide) for Thai native chicken semen [40]. Nevertheless, a lower trehalose concentration (100 mM) might have a protective effect during chicken sperm cryopreservation [47]. Mosca et al. [47] found a positive synergic action of trehalose and 6% DMA (N, N-Dimethylacetamide) on the motile function of frozen/thawed chicken sperm. The positive cryoprotective action of trehalose was on the quality of sperm motion, not on the proportion of viable and motile sperm or the recovery of progressive motile sperm after cryopreservation [49].

The use of 100 mM trehalose resulted in favorable outcomes when applied to frozen/thawed poultry semen [70]. This disaccharide was added to both cryoprotectants tested (DMA and NMA) and confirmed that both permeable cryoprotectants, together with a constant concentration of trehalose, are effective cryoprotectants that allowed the recovery of 44% of spermatozoa with intact membranes, 59% of motile spermatozoa, and 45% of progressive motile spermatozoa after cryopreservation. The cryoprotective action on sperm integrity of both permeable cryoprotectants was dose-dependent and progressively increased if the permeable cryoprotectant concentration also increased from 2 to 6%. Madeddu et al. [71] employed a modified Lake’s dilution to dilute semen before freezing. This was then supplemented with 100 mM trehalose and 2% NMA, as recommended by Zaniboni et al. [70]. The results demonstrated that this concentration enhanced the progressive motility of the sperm.

In contrast, some authors have found significant protection against freezing damage when high concentrations of trehalose (350 mM and 375 mM) were used in mouse [72] and goat [27] spermatozoa. The different results of these studies may be due to the different composition of the extenders used or different species showing different levels of tolerance to trehalose. Therefore, the effect of various concentrations of trehalose should be reevaluated in all species. Consequently, a higher concentration of trehalose was employed in the present study than that recommended by most of the aforementioned authors.

Upon verifying the selected higher concentration of trehalose (200 mM), negative results were observed in this study. This can be attributed to the increase in osmolarity of the dilute solution that occurs when sugar is added at this concentration. This, in turn, has been shown to result in a decrease in individual motility and an increase in the percentage of dead and abnormal spermatozoa. It is well established that spermatozoa motility is susceptible to changes in extracellular osmolarity [9,24,33]. The occurrence of this phenomenon can be attributed to the high osmolarity of the extender, which proved to be deleterious to the sperm cells. The osmolarity of the solution used in the extender preparation has a significant effect on the post-thaw characteristics of sperm. This finding is consistent with the observations of ram [9], which reported a negative effect on post-thaw motility and plasma membrane integrity when 200 and 400 mM trehalose were used. The concentration of trehalose in excess of 100 mM did not result in an enhancement of the post-thaw quality of bull sperm [19] or ram sperm [73]. The precise mechanism by which a high trehalose concentration exerts a negative influence on sperm quality remains unclear. However, it is thought that higher doses may alter the media’s osmolarity, thereby increasing the external pressure on sperm and altering cell architecture by inducing membrane protein denaturation, lipid phase transitions, and reducing membrane fluidity [63]. It has been demonstrated that higher concentrations of trehalose have the effect of increasing the viscosity of semen, which has been shown to result in a reduction in sperm motility. This effect has been attributed to the suppression of sperm flagella movement, although this has been partially recovered by diluting the freezing solution [4].

### 4.2. The Effect of Trehalose Supplementation on the Fertilization Ability of Spermatozoa In Vivo

It is reasonable to assume that cryopreservation of sperm with trehalose will result in reduced acrosomal damage and increased membrane fluidity. Consequently, it can be postulated that the fertility of the resulting offspring will be affected. 

To date, only a limited number of studies have investigated the potential beneficial effect of trehalose supplementation on the fertility of cryopreserved poultry semen. Mosca et al. (2019) observed encouraging results regarding the fertility rate (45% vs. 43%) when the combination of 100 mM trehalose with different concentrations of permeable cryoprotectants (DMA and NMA) was tested. The high fertilization rate is likely attributable to the favorable sperm viability and motility outcomes observed following thawing, in addition to the utilization of the Hi-Line hybrid strain, which is distinguished by its high fertility and hatchability. In a study by Madeddu et al. [71], the fertilization rate was found to be only 16.43%. This value was obtained by averaging the fertility of the three different breeds included in this experiment. In our experiment, the fertilization rate obtained with the addition of 100 mM trehalose was 23.21%. This indicates that breed also has a significant effect on ejaculate freezing. 

The purpose of this study was to assess in vitro and in vivo the effect of the addition of the impermeable disaccharide trehalose on the kinematic and functional parameters of rooster spermatozoa and their fertilization capacity after thawing. The results of the in vitro assessment of mCASA and FC in Experiment 1 indicated that there were statistically significant differences (*p* < 0.05) in the TMOT and PMOT variables. It is worth noting that the highest values were observed in the group that received the addition of 100 mM trehalose. Furthermore, the highest proportion of sperm with an intact plasma membrane and acrosome was observed in the group that received the addition of 100 mM trehalose. Differently from the in vivo assay, no significant differences were detected across evaluated trehalose concentrations. However, we clearly demonstrated trends reflecting that the in vitro assay supported a near-significant difference between TRE100 and TRE200 (*p* < 0.06). There are potential reasons for the differences between the in vivo and in vitro assays. Our in vivo assay followed the methodical approaches published by Mehdipour et al. [53], Hastuti et al. [74] or Asmarawati et al. [75], as we were able to cover the whole in vitro experiment using in vivo verification. It is possible that the number of inseminated hens or collected eggs in experimental groups may be a contributing factor to the lack of significant or near-significant differences observed, despite the findings of previous research. Therefore, it would be beneficial for future studies to consider this assumption and integrate it into their methodological design. It should be noted, however, that the results of in vitro ejaculate evaluation do not constitute an absolute indicator of the fertility of an individual in vivo. This can be attributed to the influence of numerous physiological factors present in both males and females that can impact fertility during the process of artificial insemination in breeding [76]. Two aspects of the reproductive physiology of male birds are considered to be of particular importance in influencing fertility during artificial insemination. The first of these relates to innate or physiological factors determining sperm quality, while the second concerns the method of sperm collection and processing prior to insemination. The success of insemination may be influenced by various factors associated with the procedure and the female [77]. These include the number of spermatozoa inseminated, the timing of insemination, the changing oviduct environment associated with the reproductive state of the female [78], and the development of possible immunity against spermatozoa in the female. An important factor influencing the ability to fertilize is sperm storage in the oviduct. Here, specialized invaginations of the oviductal epithelium, referred to as the sperm storage tubules, can be found in the uterovaginal junction. The tubules have the capacity to store sperm for extended periods, thus retaining its potential to fertilize. The molecular and physiological mechanisms underlying sperm storage tubule differentiation, sperm protection, and regression remain largely unknown. However, further investigation into these mechanisms may yield insights with the potential to substantially improve hen fertility, sperm storage, and semen cryopreservation in poultry [77,79].

In contrast, a notable correlation has been identified between in vitro and in vivo analyses [80,81,82]. In vitro analysis provides valuable insight into the fertilizing potential of cells, including sperm motility, which is widely regarded as the most crucial phenotypic trait associated with sperm fertilization [79]. Nevertheless, this knowledge must be supplemented by empirical data obtained from in vivo analyses, which offer a more comprehensive evaluation of the actual fertilization capacity of sperm, as demonstrated in our study.

The objective of this study was to assess the impact of the addition of the impermeable disaccharide trehalose on the functional parameters of cockerel spermatozoa and their fertilization capacity after thawing. To the best of our knowledge, there are several studies that employ flow cytometry to evaluate the functional characteristics of rooster sperm [83,84,85]. However, previous studies investigating the addition of non-permeable cryoprotectants in the cryopreservation of poultry semen have employed alternative evaluation methods, including phase contrast microscopy [7,69,70] and fluorescence microscopy [48,71]. In this study, the methodology employed for the experiment was to assess the quality of key kinematic and functional parameters of spermatozoa by combining the use of computer-assisted sperm analysis (CASA) and flow cytometry. This methodology was selected due to the lack of common practice, particularly within the field of poultry, of utilizing these techniques in conjunction. The novelty of this approach lies in the assessment of key functional and kinematic parameters of sperm in the native Czech hen breed using a combination of mCASA and flow cytometry.

## 5. Conclusions

The objective of this study was to identify the optimal cryopreservation protocol for the freezing of rooster semen by adding defined concentrations of trehalose. The potential fertility of frozen/thawed insemination doses was evaluated in vitro, with subsequent verification by in vivo insemination. We believe that these parameters, obtained using a combination of kinematic analysis and flow cytometry technology, are critical in determining the potential fertility of frozen/thawed semen. In comparison to the other test groups and the control group, the addition of 100 mM trehalose was found to significantly enhance total and progressive motility after thawing and maintained plasma membrane and acrosome integrity (*p* < 0.05). Additionally, the fertility rate indicated for the beneficial effect with 100 mM trehalose was numerically higher than with the use of semen that had been frozen using another concentration or without the trehalose addition. It is important to note, however, that this study did not yield statistically significant results with regard to the comparison of the groups under investigation. It is possible that the number of inseminated hens in the experimental groups may be a contributing factor to the lack of significant or near-significant differences observed. 

The results of this study provide valuable insights into the efficacy of in vivo and in vitro assays, offering a promising foundation for further research. The addition of the non-permeable cryoprotectant disaccharide trehalose, in particular, shows promise as a strategy for avian sperm cryopreservation. Future studies could explore the potential of trehalose to enhance the quality of rooster sperm, building upon observations made in other species regarding the impact of trehalose supplementation on antioxidant activity.

## Data Availability

The original contributions presented in the study are included in the article/Supplementary Material, further inquiries can be directed to the corresponding author.

## Supplementary Materials

The following supporting information can be downloaded at: https://www.mdpi.com/article/10.3390/ani14243586/s1, Table S1. Effect of addition of different concentrations of trehalose on the functional parameters of semen before freezing—CASA and flow cytometry evaluation (LSM ± SEM). Table S2. Effect of addition of different concentrations of trehalose on the functional parameters of semen after thawing—CASA and flow cytometry evaluation (LSM ± SEM).
