# Supplementary material for: In Vitro and In Vivo Evaluation of the Fertilization Capacity of Frozen/Thawed Rooster Spermatozoa Supplemented with Different Concentrations of Trehalose
Source: Animals (Basel). 2024 Dec 12;14(24):3586. doi: 10.3390/ani14243586 (PMC11672769; doi:10.3390/ani14243586)
Supplement: Supplementary file 1 [file animals-14-03586-s001.zip › animals-3272531-supplementary.pdf]

Table S1. Effect of addition of different concentrations of trehalose on the functional parameters of semen before freezing—CASA and flow cytometry evaluation (LSM ± SEM).

Variable	Trehalose Supplementation				Significance	
	C (n=30)	TRE50 (n=18)	TRE100 (n=30)	TRE200 (n=18)	DAY	TRE
**TMOT (%)**	73.60±1.413^{ac}	73.16±1.965^{a}	70.70±1.413^{ab}	65.77±1.965^{bc}	****	**
**PMOT (%)**	16.40±1.212^{a}	13.49±1.568^{a}	15.10±1.212^{a}	16.06±1.968^{a}	****	n.s.
**VCL (μm/s)**	78.80±1.255^{a}	76.92±1.769^{a}	75.83±1.255^{a}	76.29±1.769^{a}	****	n.s.
**VAP (μm/s)**	47.48±0.958^{a}	46.34±1.348^{a}	48.03±0.958^{a}	47.96±1.348^{a}	****	n.s.
**VSL (μm/s)**	34.30±0.938^{a}	30.76±1.332^{a}	32.20±0.938^{a}	32.86±1.332^{a}	****	n.s.
**STR (%)**	48.67±0.911^{a}	44.34±1.278^{a}	46.73±0.911^{a}	48.42±1.278^{a}	****	*
**LIN (%)**	61.90±0.979^{a}	58.54±1.366^{a}	60.57±0.979^{a}	58.98±1.366^{a}	****	n.s.
**PI^-/FITC-PNA^- (%)**	68.04±2.142^{ac}	72.44±2.945^{a}	71.19±2.101^{a}	59.79±2.949^{bc}	****	**
**PI^+/FITC-PNA^- (%)**	28.23±1.934^{b}	24.04±2.652^{b}	25.44±1.891^{b}	37.17±2.652^{a}	****	**
**PI^-/FITC-PNA^+ (%)**	3.31±0.462^{a}	3.17±0.631^{a}	2.78±0.452 ^{a}	2.61±0.631^{a}	****	n.s.
**PI^+/FITC-PNA^+ (%)**	0.48±0.082^{a}	0.35±0.111^{b}	0.59±0.082^{a}	0.42±0.111^{a}	****	n.s.

Abbreviations: C – control group (no added trehalose); TRE50 – samples containing 50 mM of trehalose; TRE100 – samples containing 100 mM of trehalose; TRE200 – samples containing 200 mM of trehalose; DAY – effect of fixed day semen collection; TRE – effect of fixed addition of cryoprotective media containing different concentrations of trehalose; TMOT – total motility; PMOT – progressive motility; VAP – average path velocity; VCL – curvilinear velocity; VSL – linear velocity; LIN – linearity; STR – straightness; PI^-/FITC-PNA^- – spermatozoa population with negative signal of plasma membrane or acrosome disruptions located in the left lower quadrant; PI^+/FITC-PNA^- – spermatozoa population with damaged plasma membrane and slightly deteriorated acrosome located in the left upper quadrant; PI^-/FITC-PNA^+ – spermatozoa population with percentage of sperm with high acrosome damage and intact plasma membrane located in the right lower quadrant; PI^+/FITC-PNA^+ – spermatozoa population with high damage to the plasma membrane and acrosome located in the right upper quadrant. Level of significance: n.s. - not significant = (P >0.05), * = (P <0.05), ** = (P <0.01), **** = (P <0.0001). a-b Different letters indicate differences between groups within a row (P < 0.05).

Table S2. Effect of addition of different concentrations of trehalose on the functional parameters of semen after thawing—CASA and flow cytometry evaluation (LSM ± SEM).

Variable	Trehalose Supplementation				Significance	
	C (n=30)	TRE50 (n=18)	TRE100 (n=30)	TRE200 (n=18)	DAY	TRE
TMOT (%)	26.57±0.902[b]	16.40±1.264[b]	34.17±0.902[a]	16.65±1.264[b]	****	****
PMOT (%)	2.73±0.406[b]	1.63±0.570[b]	3.57±0.406[a]	1.99±0.600[b]	**	*
VCL (µm/s)	62.17±1.566[a]	58.70±2.193[a]	64.53±1.566[a]	61.52±2.193[a]	***	n.s.
VAP (µm/s)	38.40±0.961[a]	37.70±1.346[a]	39.07±0.961[a]	37.70±1.346[a]	*	n.s.
VSL (µm/s)	25.03±1.021[a]	25.13±1.428[a]	25.27±1.021[a]	23.70±1.428[a]	n.s.	n.s.
STR (%)	51.93±1.961[a]	58.90±2.746[a]	50.43±1.961[a]	51.15±2.746[a]	****	n.s.
LIN (%)	52.73±1.919[a]	50.42±2.687[a]	51.07±1.919[a]	47.31±2.687[a]	****	n.s.
PI^-/FITC-PNA^- (%)	35.05±1.194[a]	30.74±1.854[b]	33.37±1.194[a]	29.05±1.673[b]	****	*
PI^+/FITC-PNA^- (%)	57.64±1.364[a]	63.62±2.119[a]	60.42±1.364[a]	61.86±1.911[a]	****	n.s.
PI^-/FITC-PNA^+ (%)	6.87±0.637[a]	5.97±0.989[a]	5.73±0.637[a]	7.20±0.892[a]	****	n.s.
PI^+/FITC-PNA^+ (%)	0.44±0.111[a]	0.18±0.173[b]	0.48±0.111[a]	0.83±0.156[a]	****	***

Abbreviations: C – control group (no added trehalose); TRE50 – samples containing 50 mM of trehalose; TRE100 – samples containing 100 mM of trehalose; TRE200 – samples containing 200 mM of trehalose; DAY – effect of fixed day semen collection; TRE – effect of fixed addition of cryoprotective media containing different concentrations of trehalose; TMOT – total motility; PMOT – progressive motility; VAP – average path velocity; VCL – curvilinear velocity; VSL – linear velocity; LIN – linearity; STR – straightness; PI^-/FITC-PNA^- – spermatozoa population with negative signal of plasma membrane or acrosome disruptions located in the left lower quadrant; PI^+/FITC-PNA^- – spermatozoa population with damaged plasma membrane and slightly deteriorated acrosome located in the left upper quadrant; PI^-/FITC-PNA^+ – spermatozoa population with percentage of sperm with high acrosome damage and intact plasma membrane located in the right lower quadrant; PI^+/FITC-PNA^+ – spermatozoa population with high damage to the plasma membrane and acrosome located in the right upper quadrant. Level of significance: n.s. - not significant = ($P > 0.05$), * = ($P < 0.05$), ** = ($P < 0.01$), *** = ($P < 0.001$), **** = ($P < 0.0001$). a-b Different letters indicate differences between groups within a row ($P < 0.05$).
